# Supplementary material for: Content-rich biological network constructed by mining PubMed abstracts
Source: BMC Bioinformatics. 2004 Oct 8;5:147. doi: 10.1186/1471-2105-5-147 (PMC528731; doi:10.1186/1471-2105-5-147)
Supplement: Additional File 5 — The original Chilibot query results of the term "long-term potentiation (LTP)" and 22 other terms, limiting the latest references analyzed to the years 1990, 1995, 2000, and 2004. [file 1471-2105-5-147-S5.bz2 › chilibotAdditionalFile5/ltp2004/html/NMDA_CAMKIV.html]

 


 **NMDA** and **CAMKIV** 
  
Found 3 abstracts in PubMed,  **3 abstracts were retrieved and analyzed**.  


---

 Search Google  |
 PDF files only 
|  EDU domain only 

---

**Interactive relationship** (e.g. stimulation, inhibition, etc)

- We show that  **NMDA**  activates transcription of CREB responsive genes in hippocampal neurons, with ERK responsible for persistent CREB phosphorylation and CaM kinase IV  **CaMKIV**  responsible for phosphorylating the CREB coactivator, CBP.  Ref: 11970865 Neuron, 2002
- Several effects were demonstrated sustained exposure to 25 mM KCl or 140 microM  **NMDA**  produced  **CaMKIV**  down regulation, compared to 5 mM KCl cultures.  Ref: 12064479 J Neurochem, 2002

**Parallel relationship** (e.g. studied together, co-existance, homology, etc.)

- Additionally, mutation of Ser301 impaired  **NMDA**  and  **CaMKIV**  stimulated transcription.  Ref: 11970865 Neuron, 2002
